# SOCS2 Influences LPS Induced Human Monocyte-Derived Dendritic Cell Maturation

**DOI:** 10.1371/journal.pone.0007178

**Published:** 2009-09-25

**Authors:** Jin Hu, Ola Winqvist, Amilcar Flores-Morales, Ann-Charlotte Wikström, Gunnar Norstedt

**Affiliations:** 1 Department of Molecular Medicine and Surgery, Karolinska Institute, Stockholm, Sweden; 2 Unit of Clinical Allergy Research, Department of Medicine Solna, Karolinska Institute, Stockholm, Sweden; 3 Department of Biosciences and Nutrition, Karolinska Institute, Stockholm, Sweden; 4 Department of Trauma Orthopedic & Hand Surgery, The First Affiliated Hospital of Guangxi Medical University, Guangxi Medical University, Nanning, Guangxi, P.R. China; New York University School of Medicine, United States of America

## Abstract

Dendritic cells (DCs) are highly specific antigen presenting cells, which link innate and adaptive immune responses and participate in protecting hosts from invading pathogens. DCs can be generated *in vitro* by culturing human monocytes with GM-CSF and IL-4 followed by LPS induced DC maturation. We set out to study the suppressor of cytokine signaling (SOCS) proteins during maturation and activation of human monocyte-derived DCs from peripheral blood *in vitro*. We found that the expression of SOCS2 mRNA and protein is dramatically up-regulated during DC maturation. Silencing of SOCS2 using siRNA, inhibited DC maturation as evidenced by a decreased expression of maturation markers such as CD83, co-stimulatory molecules CD40, CD86 and HLA-DR. Furthermore, silencing of SOCS2 decreased LPS induced activation of MAP kinases (SAKP/JNK, p38, ERK), IRF3, decreased the translocation of the NF-κB transcription factor and reduced downstream gene mRNA expression. These results suggest a role for SOCS2 in the MyD88-dependent and -independent TLR4 signaling pathways. In conclusion, our results demonstrate that SOCS2 is required for appropriate TLR4 signaling in maturating human DCs via both the MyD88-dependent and -independent signaling pathway.

## Introduction

The innate immune system is the first line of defense protecting the host from invading pathogens. Dendritic cells (DCs) serve as highly specific APCs and play a crucial role connecting the induction of innate immunity and the subsequent development of the adaptive immune response [1], [2]. In this process, DC maturation serves as the critical switch from maintenance of self-tolerance to the induction of immunity [3]. Mature DCs increase the expression of co-stimulatory molecules, as well as MHC I and II and diverse immune regulative molecules that stimulate naive Th cells to differentiate into Th1 or Th2 cells [4], [5]. DCs also secrete large amounts of pro-inflammatory cytokines that activate innate lymphocytes to kill infected cells that have been invaded by pathogens [4], [6].

DC's recognize pathogen-associated molecular patterns by various pattern recognition receptors. Among these receptors, TLRs expressed on APCs, such as DCs and macrophages, serve as key pattern recognition receptors [7]. There are 11 human and 13 mouse TLRs identified to date, and each TLR member precisely recognizes distinct pathogen-associated molecular patterns derived from various microorganisms and activate inflammatory cytokines, chemokines, IFNs and upregulate the expression of co-stimulatory molecules [1]. LPS is a gram negative bacterial cell wall component and a TLR4 ligand [8], [9]. Ligand-induced dimerization activates the TLR4, and adapters are recruited via their Toll-interleukin 1 receptor (TIR) domains. MyD88 is a universal adaptor and acts to recruit the interleukin 1-associated kinas (IRAK) family, TNF receptor-associated factor (TRAF) 6 and IκBα kinases which leads to the activation of the transcription factor, NF-κB and also MAP kinases (JNK, p38, ERK) [10]. MyD88-adaptor like (MAL) is also recruited by TLR4 and stabilize MyD88 in the complex [10]. The above pathway is termed the MyD88-dependent pathway. In addition the MyD88-independent signaling pathway activates a TIR domain-containing adaptor (TRIF), which needs another bridge adaptor, the TRIF-related adaptor molecule (TRAM), and this leads to activation of TRAF3 that contributes to the activation of interferon regulatory factor (IRF) 3 [10], [11] and the late phase activation of NF-κB and MAP kinases [12].

Monocytes have been shown to be important DC precursor cells both in vitro and in vivo [13], [14]. Monocyte-derived DCs can be generated by monocyte cultivation with GM-CSF and IL-4 [15], [16] or IL-13 [17] in vitro, and this makes it possible to generate large quantities of DCs providing a model to investigate the effect of self or environmental agents on the differentiation and maturation pathways of DCs.

Suppressor of cytokine signaling (SOCS) family includes eight members, characterized by the presence of a Src homology 2 domain and a C-terminal conserved domain called the SOCS box [18], Each family member plays a unique role in attenuating cellular signaling [19], [20]. SOCS1 and SOCS3 have recently been demonstrated to negatively regulate TLR signaling in macrophage and DC maturation [21], [22], [23]. Although SOCS2 is a well known negative regulator of some signaling pathways such as the JAK/STAT pathway [24], there is little knowledge about the role of SOCS2 in TLR signaling. One study has demonstrated that SOCS2 mRNA expression increased during differentiation and maturation of mouse DCs [25], which suggested a possible SOCS2 involvement in DC function, but subsequent over-expression of the SOCS2 protein did not influence TLR signaling in mouse macrophages [26]. Another study showed that SOCS2 might be involved in the regulation of the immune response upon infection. SOCS2 mRNA and protein were induced by lipoxin (LXA4), an eicosanoid mediator with potent anti-inflammatory properties in DCs, and SOCS2 knock-out mice showed decreased microbial proliferation, leukocyte infiltration, production of pro-inflammatory cytokines, and a high mortality upon infection [27]. These findings suggest that SOCS2 may have an important role in the immune response to diverse infectious agents.

In this study, we generated DCs from human monocytes cultured with GM-CSF and IL-4 and used them to investigate the role of SOCS proteins and TLR4 signaling pathways in DC maturation.

## Results

### Gene expression of SOCS family members during maturation of human monocyte-derived DCs

**Figure 1:**
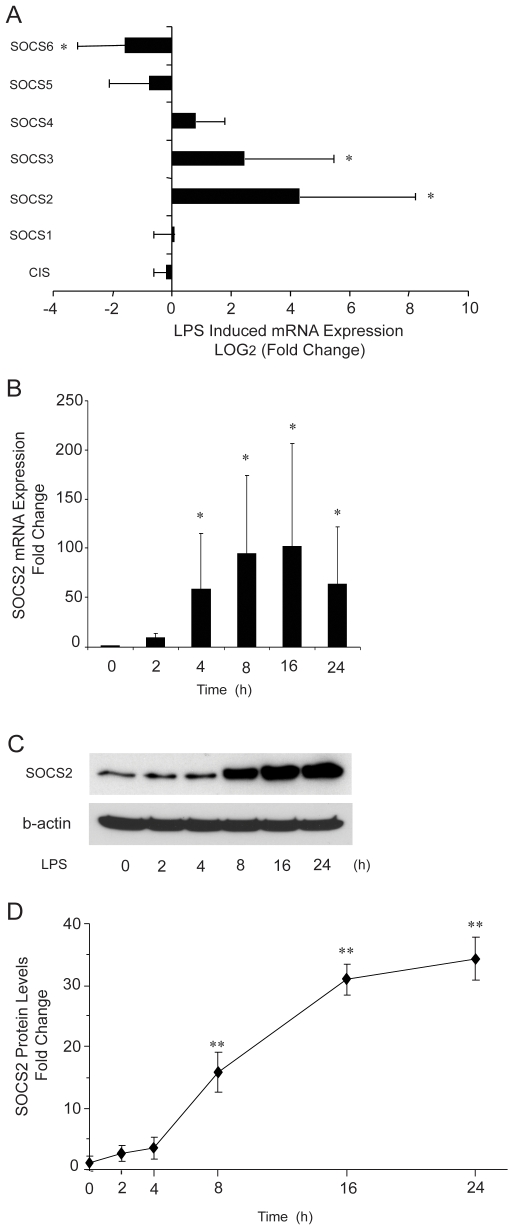
Expressions of SOCS family members during human monocyte-derived DC maturation. (A). mRNA expression of SOCS family members during DC maturation. Enriched monocytes were cultured in the presence of GM-CSF and IL-4 for 6 days. Quantitative real-time PCR was used to measure SOCS mRNA expression in iDC at d6 and at d7, after exposure to LPS for 24 h. Data shown are the mean of duplicate determination from four donors, fold changes were obtained by comparing effects before and after of LPS treatment. (B, C, D). SOCS2 mRNA and protein expression levels during iDC maturation. Quantitative real-time PCR for mRNA and Western Blotting analysis for protein of SOCS2 expression in iDCs exposed to LPS for different time periods. The data shown in (B) are the mean of triplicate determination from four donors. Values for 0 time point were set to 1. The picture shown in (C) represents one Western Blot from a typical donor sample. The data shown in (D) are the mean of SOCS2 bands quantified from Western blots and normalized to β-actin from four donors. Values for 0 time point were set to 1. Error bars illustrate s.d. * P<0.05; ** P<0.01.

Previous studies have shown that cytokine-inducible SH2-domain protein (CIS), SOCS1, SOCS2 and SOCS3 gene expressions are regulated by LPS stimulation in mouse DCs or macrophages [25], [26]. In addition, IL21 [28] and protein allergens [29] can change expressions of SOCS 1 and SOCS 3 in humans DCs. We first studied the gene expression of SOCS members during DC maturation. Human monocyte-derived immature DCs (iDCs) were obtained by culturing with GM-CSF and IL-4. Subsequently the cells were treated with LPS at day 6 of differentiation and harvested for mRNA measurement after 24 h. As shown in Fig. 1A, SOCS2, SOCS3 and SOCS6 changed significantly after LPS treatment, whereas the other SOCS family members CIS, SOCS1, 4 and 5 did not change in expression levels. Based on the regulatory changes of SOCS2 mRNA expression in our experiment, we decided to further focus on the role of SOCS2 in the process of DC maturation. Real-time PCR analysis of total RNA revealed that SOCS2 mRNA levels start to increase at 2 h after LPS treatment and became significant from 4 h till 24 h (Fig. 1B). Western Blotting analysis of whole cell protein extracts showed that also SOCS2 protein levels increased after LPS treatment in a manner that corresponded to the changes seen in mRNA levels. The effect was weak at 4 h, but became significant at 8 h and increased dramatically up to 16 h where the expression was sustained for at least 24 h (Fig. 1C, D).

### SOCS2 influences MyD88-dependent and MyD88-independent signaling in the TLR4 signaling pathway

**Figure 2:**
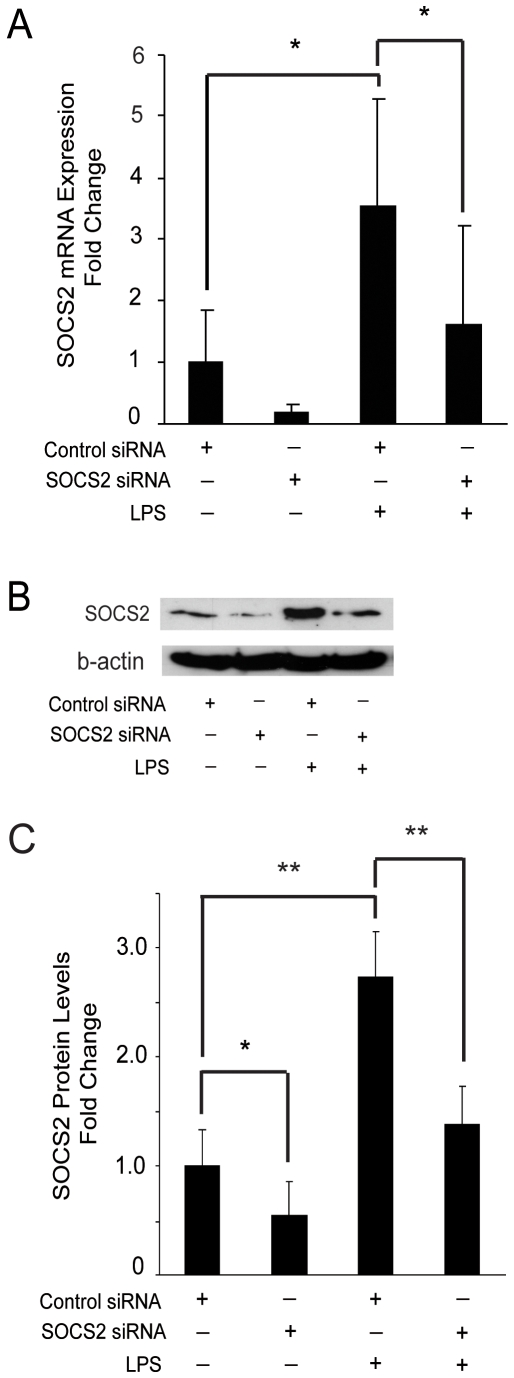
SOCS2 siRNA inhibits SOCS2 mRNA and protein expression in DCs during maturation. Enriched monocytes were cultured in the presence of GM-CSF and IL-4 for 6 days. iDCs were transfected with SOCS2 siRNA or control siRNA and incubated for 24 hours. (A). SOCS2 mRNA gene expression from DC transfected with SOCS2 silencing RNA during maturation. Transfected cells were exposed to LPS (200 ng/ml) for 2 hours, the cells were then harvested for the measurement of SOCS2 RNA expression by qRT-PCR. Data shown were mean of duplicate determination from 4 donors. (B, C). SOCS2 protein level expression from DCs transfected with SOCS2 silencing RNA during maturation. Transfected cells were exposed to LPS (200 ng/ml) for 8 hours, after which whole-cell lysates were extracted for the measurement of SOCS2 protein levels by Western Blotting. (B) Picture shown is 1 of 4 typical donors. (C) Data shown are mean of SOCS2 bands quantified and normalized to β-actin from four donors. Values for the control were set to 1. Error bars illustrate s.d. * P<0.05; ** P<0.01.

LPS affects iDC function through activation of the TLR4 signaling pathway, which downstream consists of the MyD88-dependent and the TRIF-dependent pathway, the latter is also called the MyD88-independent pathway [11]. First, we wanted to test whether SOCS2 induced by LPS can affect the TLR4 signaling pathway. A specific silencing RNA (siRNA) targeting human SOCS2 or negative control siRNA with no homology to any known mammalian gene were transfected into iDC, and SOCS2 mRNA expression was then monitored. Low expression levels of SOCS2 was detected in untreated DCs, and as expected LPS treatment induced SOCS2 expression in control cells, an effect that was significantly reduced in SOCS2 siRNA-transfected cells both at the level of mRNA (Fig. 2A) and protein (Fig. 2B, C).

**Figure 3:**
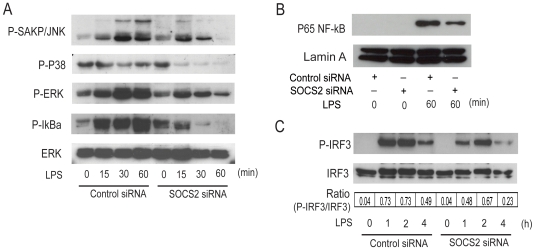
SOCS2 siRNA inhibits TLR4 signaling of DC via inhibition of both MyD88-dependent (A, B) and -independent pathway (C). Enriched monocytes were cultured in the presence of GM-CSF and IL-4 for 6 days. iDCs were transfected with SOCS2 siRNA or control siRNA and incubated for 24 hours. (A). SOCS2 knock-down effect on phosphorylation of SAKP/JNK, P38, ERK and IκBα. Transfected cells were stimulated with LPS at the indicated time points. The level of phosphorylation of SAKP/JNK, P38, ERK and IκBα was determined by Western Blotting of whole-cell lysates using antibodies specific for the phosphorylated, activated forms of the proteins. The levels of total ERK was determined as loading controls. (B). SOCS2 knock-down effect on nuclear translocation of NF-κB. Transfected cells were stimulated with LPS at the indicated time points. Nuclear protein was extracted for Western Blotting measurement. The level of P65 NF-κB and Lamin A (as loading control) was detected. (C). SOCS2 knock-down effect on phosphorylation of IRF3. Transfected cells were stimulated with LPS at the indicated time points. The level of phosphorylated and total IRF3 was determined by Western Blotting of whole-cell lysates using antibodies specific for the forms of the proteins, respectively. The IRF3 bands were quantified, and the ratio of phosphorylated to total IRF3 were shown.

To study the effect of SOCS2 on the MyD88-dependent signaling pathway, proteins from control or SOCS2 siRNA transfected DCs were extracted. LPS activation of this pathway results in a cascade of kinase dependent phosphorylations. The kinetics of the phosphorylation of SAKP/JNK, P38, ERK and IκBα was measured by Western Blotting. In cells with SOCS2 siRNA, LPS treatment led to a reduced phosphorylation of SAKP/JNK, P38, ERK and IκBα (Fig. 3A). When nuclear proteins were extracted from control or SOCS2 siRNA treated DCs the SOCS2 silenced DCs displayed a weaker NF-κB band compared to control DCs (Fig. 3B). This indicates that less NF-κB was translocated into the nucleus after SOCS2 elimination, supporting the notion that SOCS2 silencing inhibits the MyD88-dependent signaling pathway in human DCs by interrupting the normal SOCS2 dependent kinase cascade.

Protein extracts from control or SOCS2 siRNA transfected DCs were then tested for the effect of SOCS2 on the MyD88-independent signaling pathway. IRF3, a kinase that is phosphorylated and translocated into the nucleus following LPS stimulation, was phosphorylated to a lesser extent in the SOCS2 knock-down DCs (Fig. 3C). This supports the notion that TLR4 signaling is SOCS2 dependent in human DCs, not only via the MyD88-dependent but also via the MyD88-independent signaling pathway. In initial studies, we tested two different SOCS2 siRNAs having different sequences. These experiments showed that there were no differences in the extent of SOCS2 reduction and effect between the two SOCS2 siRNAs. For this reason we selected one SOCS2 siRNA in subsequent experiments.

### LPS induced gene expression is dependening on SOCS2

**Figure 4:**
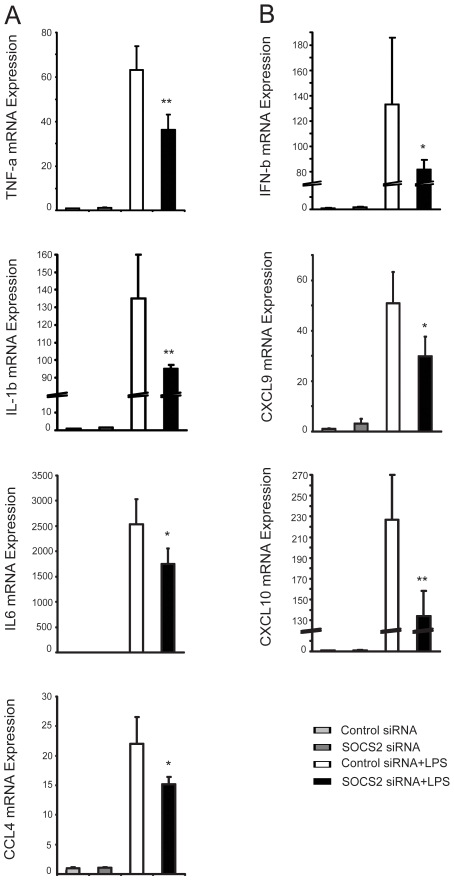
SOC2 siRNA affects LPS inducible gene expression both in the MyD88-dependent and -independent signaling pathway. Enriched monocytes were cultured in the presence of GM-CSF and IL-4 for 6 days. iDCs were transfected with SOCS2 siRNA or control siRNA and incubated for 24 hours. Cells were further incubated with LPS (200 ng/ml) for additional 2 h. LPS inducible gene mRNA expressions were measured by real-time PCR. (A). LPS inducible gene expression of TNF-α, IL-6, IL-1β and CCL-4 via the MyD88-dependent signaling pathway. (B) LPS inducible gene expression of IFN-β, CXCL-9, CXCL-10 via the MyD88-independent signaling pathway. Data shown are the mean of triplicate sample, and are representative of at least two donors with similar results. Values for the control were set to 1. Error bars illustrate s.d. * P<0.05; ** P<0.01.

Activation of the TLR4 signaling pathway induces, for example, TNF-α, IL-6, IL-1β and CCL-4 via the MyD88-dependent pathway [30], [31] and IFN-β, CXCL-9, and CXCL-10 via the MyD88-independent pathway [32], [33]. To investigate if the inhibition of TLR4 signaling by SOCS2 silencing caused subsequent changes in mRNA expression levels of downstream mediators in this pathway, we measured LPS inducible gene expression by quantitative PCR. SOCS2 knock-down significantly decreased mRNA expression of TNF-α, IL-6, IL-1β and CCL-4 (Fig. 4A), and similar effects were seen on the expression of INF-β, CXCL-9, and CXCL-10 (Fig. 4B). These findings suggests that SOCS2 influences LPS inducible gene expression via both the MyD88-dependent and independent signaling pathway.

### Role of SOCS2 in DC maturation in response to inflammatory signals

**Figure 5:**
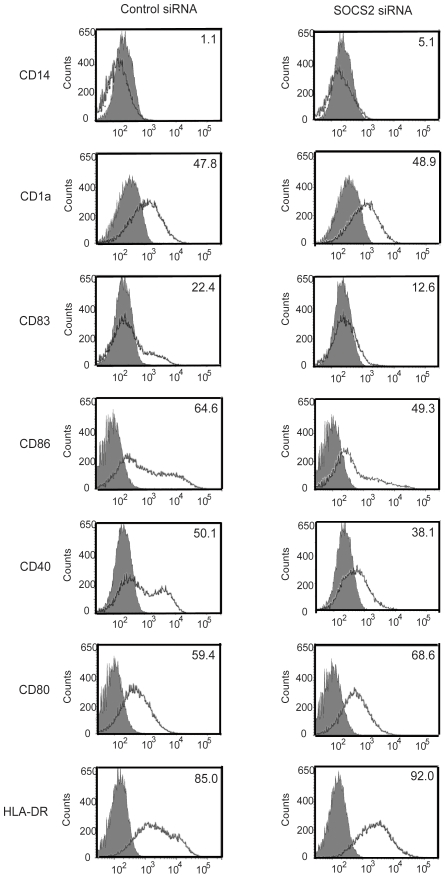
Effect of SOCS2 siRNA in DC maturation. Enriched monocytes were cultured in the presence of GM-CSF and IL-4 for 6 days. iDCs were transfected with SOCS2 siRNA or control siRNA and incubated for 24 hours. Histograms showing the effect of SOCS2 knock-down on DC maturation. LPS (200 ng/ml) was added and were present for 24 hours. Afterwards, cells were harvested, stained, and analyzed by flow cytometry. The histogram shows changes of indicated surface molecules in DCs from the different groups. Matched isotype controls are presented as solid histograms. Percentage of positive cells is indicated in the upper right corner of each histogram.

As the LPS effect on DC maturation is mediated via TLR4 signal transduction we decided to investigate a possible role of SOCS2 in DC maturation. SOCS2 knock-down DCs were stimulated with LPS 24 h after siRNA transfection and harvested for flow cytometry analysis. LPS stimulation of SOCS2 siRNA transfected iDCs led to an impairment in DC maturation. This was demonstrated by changes in surface molecule expression. CD83, the marker for mature DCs [34], was significantly decreased in SOCS2 siRNA transfected DCs. Moreover, these DCs showed a weaker expression of the co-stimulatory molecules CD40, CD86, whereas no evident effect on CD80 and HLA-DR was demonstrated, based on the percentage of positive cells (% positive cells) compared with control group (Fig. 5). Cells transfected with SOCS2 siRNA showed a decrease in mean fluorescence intensity around 40%, for the CD83 marker and 39%, for the HLA-DR marker, as compared to the levels in control cells (data no shown).

## Discussion

Our data confirm and further extend the knowledge of SOCS involvement in DC maturation [25], [26] and indicate that SOCS2 is an important SOCS family member involved in the maturation process. This was demonstrated by a clear increase of both SOCS2 mRNA and protein during DC maturation and the finding that SOCS2 silencing leads to an impaired maturation of DCs. LPS stimulation of iDCs where SOCS2 had been silenced failed to express CD83, a typical marker of DC maturation. In addition, such cells showed a lower expression of the co-stimulatory molecules CD40, CD86 and HLA-DR. We showed that SOCS2 silencing decreased both MyD88-dependent and -independent signaling in the TLR4 signaling pathway. This finding was corroborated by a corresponding decrease of LPS inducible genes encoding the effector cytokines TNF-α, IL-6, IL-1β and CCL-4. A SOCS2 dependency was also demonstrated for gene expression of INF-β, CXCL-9 and CXCL-10 i.e. genes related to the MyD88-independent signaling pathway. LPS stimulation under conditions of SOCS2 knock-down resulted in a decreased expression of all of these genes. Our results were substantiated at the protein level by Western Blots of protein extracts. Down-regulation of SOCS2 led to a decrease in NF-κB nuclear translocation in response to LPS stimulation [35] and also the phosphorylation of IRF3 [36] and the activation of MAP kinases [37] was reduced in LPS stimulated cells where SOCS2 had been silenced. Thus, SOCS2 regulates TLR signaling via the MyD88-dependent and -independent signaling pathway.

SOCS2, as other members of this family is thought to function as a subunit of an E3 ubiquitin ligase complex [38]. The SOCS box, placed at the C-terminus serves to interact with Elongins C and B, cullin5 and Rbx2 forming the catalytic core of the complex [39], [40]. The SH2 domain of SOCS2 serves to interact with phosphotyrosine residues the targeted substrate directing the specificity of the ubiquitin ligase reaction [39]. For other members of the family it has been demonstrated that SOCS targets are polyubiquitinated and subsequently degraded by the proteasome [40], [41]. Consequently, the most common effect of SOCS proteins is to reduce signaling. In dendritic cells, this is the case with SOCS3 and SOCS1, which have been shown to inhibit TLR signaling targeting TRAF6 in the case of SOCS3 [42] and MAL in the case of SOCS1 [43]. In contrast, SOCS2 elimination reduce LPS induced signals suggesting that negative regulators of TLR4 signaling are themselves targeted for degradation by SOCS2. Such negative regulators should themselves be tyrosine phosphorylated in order to interact with SOCS2. Interestingly, SOCS2 can control levels of other SOCS proteins such as SOCS3 and SOCS1 [44], and such interactions may explain why LPS signaling is diminished in the absence of SOCS2. Other SOCS proteins are not the only possible targets and proteins such as DNAX-activating protein 12 [45] and the Tyro3/Axl/Mer family of receptor kinases [46] have been shown both to be tyrosine phosphorylated as well as to act as negative regulators of TLR signaling. Interestingly, both of these pathways target MAL an early signaling intermediaries in the TLR signaling cascade. Because both the MAL/MyD88 and the TRAM-TRIF pathways are both affected by SOCS2 knock-down, it seems plausible that SOCS2 targets act early on the signaling pathway, in line with possible actions of SOCS1 or the Tyro3/Axl/Mer receptor family. In a more complex scenario, it is possible that SOCS2 has separate targets for MyD88 dependent and independent pathways. In this case several negative regulators such as Src homology 1-containing tyrosine phosphatase 1 which targets IRAK1 [47], Src homology 2-containing tyrosine phosphatase which targets TANK binding kinase 1 [48] and mitogen-activated protein kinase phosphatase-1 which targets p38MAP kinase [49] mention some of the possibilities. Obviously this is a complex problem that requires a careful examination in future studies. It is important to note that SOCS2 is known to have stimulatory activity on certain cytokine signaling pathways. It can increase the action of IL-6, an effects that has been attributed to SOCS2 reversing the inhibitory action of SOCS1 on IL-6 signaling [50]. This phenomenon may also affect the activity of cytokines produced during dendritic cell maturation that have a positive feed back on the process.

Animal experiments support a role for SOCS2 in immune regulatory pathways. Mice where the SOCS2 gene has been ablated demonstrate an increased inflammation and mortality during T.gondii infection [27]. However, LPS treatment of splenic DCs of SOCS2 knock out mice did not influence proinflammatory cytokine expression (TNF, IL12 p40, IL6 and IFN-α). Similarly, LPS could not change baseline levels of MAP kinases family, but caused a slight decrease in IκBα [51]. The reason why DC cells from SOCS2 knock out animals seem to respond differently from human cells is unclear. The fact that mature DCs in mice instead of immature DCs in humans were treated with LPS might explain the absence of LPS reactivity in DCs from SOCS2 knock out mice. Alternatively, it may exists a functional difference between human and mouse DCs regarding SOCS2.

In conclusion, our results indicate that SOCS2 positively regulates maturation of human DCs *in vitro*. This is achieved by promotion of TLR4 signaling through both the MyD88-dependent and -independent signaling pathway.

## Materials and Methods

### Ethics Statement

The procedure of human PBMCs isolation was performed following approval by the Ethics Committee of the Karolinska Institute and Karolinska University Hospital.

### Cell culture media and reagents

L-glutamine, penicillin, streptomycin and FCS (Hyclone, Logan, UT); Ficoll-Paque (Pharmacia Biotech, Uppsala, Sweden); RPMI-1640 cell culture medium and LPS derived from Escherchia coli O26: B6 (Sigma-Aldrich, St Louis, USA); Recombinant human GM-CSF and recombinant human IL-4 (Invitrogen Biosource, Camarillo, CA); and CD14^+^ Human monocyte isolation kit II (Miltenyi Biotec, Bergisch Gladbach, Germany) were purchased from the sources indicated.

### Generation of human monocyte-derived DCs

Human PBMCs were isolated from fresh heparinized blood buffy coats (not older than 8 hours) from healthy donors provided from the Department of Transfusion Medicine, Karolinska University Hospital, by a standard procedure of Ficoll-Paque density gradient centrifugation. CD14^+^ cells were negatively selected by MACS, according to the manufacturer's instructions. The mean purity of the obtained CD14^+^ cells was more than 95%, as revealed by flow cytometry. CD14^+^ cells were subsequently cultured in cell culture flasks (10^6^cells/ml) and in complete RPMI1640 medium supplemented with 10% heat-inactivated FCS, 2 mM L-glutamine, 100U penicillin and 100U streptomycin/ml, 50 ng/ml GM-CSF and 20 ng/ml IL-4 for 6 days. The cells were fed with fresh medium (half original medium volume) containing 100 ng/ml GM-CSF and 40 ng/ml IL-4 on days 2 and 4. Mature DCs were obtained from immature DCs (5×10^5^/ml), cultivated for 6 days as described above, by stimulation with 200 ng/ml LPS for 24 h after a first incubation for 2 h in fresh supplemented RPMI 1640 medium with 50 ng/ml GM-CSF and 20 ng/ml IL-4.

### Transfection

Transfection of immature DCs (iDCs) was performed using a commercial kit and a nucleofector machine (Amaxa Co., Köln, Germany). According to the manufacturer's instruction, the iDCs were collected on day 6. 10^7^ cells were resuspended in 100 µl human DC nucleofection solution. Small interfering RNA (siRNA) (Qiagen, sequences in Table S1, The control siRNA and the SOCS2 siRNAs were tested in control experiments for effects on mRNA expression of IFNβ and IFNα1 measured by Real-time PCR. In a time course study we could not observe any effect on IFNα1 and IFNβ, whereas a robust induction was seen by exposing cells to Poly I∶C. IFNβ expression was slightly increased by the transfection method however this increase was marginal compared to the effect of LPS on IFNβ expression.) was added, and the mixed samples were transferred into certified cuvettes and transfected by using program U002. 500 µl pre-warmed RPMI 1640 medium, supplemented with 10% FCS, L-glutamine, penicillin and streptomycin, was added to each cuvette after transfection. The transfection efficiency of siRNA was measured with Alexa Fluor 488 labeled negative control and Alexa Fluor 488 labeled SOCS2 siRNA and FACS, and it is shown to be near 100% in our experiments (data no shown). The transfected cells were collected and seeded into wells of 6-well plates containing supplemented RPMI 1640 medium with 50 ng/ml GM-CSF and 20 ng/ml IL-4. After 24 hours, the cells were washed and divided into several dishes for stimulation and analysis.

### Real-time PCR

Total RNA was extracted from 1−3×10^6^ cells using TRIzol (Invitrogen). RNA (1 µg) was treated with DNase I (Promega, Madison, WI) and then reverse-transcribed with a cDNA synthesis kit (Bio-Rad, Hercules, CA). The synthesized cDNA was used as template in a real-time PCR mix according to the manufacturer's standard protocol (iQ SYBR Green supermix reagents and iQ5 detection system from Bio-Rad, Hercules, CA). The reactions were performed in 20 µl with 2 µl of respective cDNA sample. As a control for the specificity of the real-time PCR a sample without template was included. For each studied gene, a relative standard curve was constructed by serial dilutions using a pool of all cDNA samples. All the measurements were performed in duplicate or triplicates for each sample and normalized to the housekeeping genes RP-II. All primer sequences are provided in Table S2.

### Flow cytometry

Antibodies for staining of human DCs were CD14 (FITC; M5E2), HLA-DR (PerCP; L243), CD 40 (APC; 5C3), CD 80 (PE; L307.4), CD 83 (PE; HB15), CD 86 (APC; B70) (BD Pharmingen, NJ, USA) and CD1a (Pacific blue; HI149) (Bioscience, San Diego, CA). Approximately 5×10^5^ DCs were harvested and stained with antibodies for 30 minutes according to standard protocols. After the incubation, the cells were washed with FACS buffer and fixed in diluted FACS lysing solution 10 minutes and finally suspended in 500 µl of FACS buffer. For multiple colors marker staining, the cells were incubated with the combination of FITC-conjugated CD14, Pacific Blue-conjugated CD1a, PE-conjugated CD83 and APC-conjugated CD86, alternatively with APC-conjugated CD40, PE-conjugated CD80 and PerCP-conjugated HLA-DR. Thirty thousand cells were acquired and analyzed on a FACS Arial flow cytometer (BD Biosciences, Heidelberg, Germany). An appropriate gate was set on the basis of scatter properties for excluding dead cells, and only cells within this gate were analyzed. Cells exhibiting a higher mean fluorescence intensity value than cells stained with respective isotype control were considered positive.

### Nuclear and cytoplasmic extraction and whole-cell lysates

iDCs were resuspended in complete RPMI 1640 medium and treated with LPS for different periods. After washing once with cold PBS, nuclear and cytoplasmic protein were extracted from 5×10^6^ DCs using a commercial nuclear and cytoplasmic protein extraction kit (Pierce Biotechnology, Rockford, IL) according to the manufacturer's instruction. To obtain whole protein lysates, cells were incubated with radio immune precipitation assay buffer (containing 100 µl/ml of a protease inhibitor cocktail solution [Roche, Penzberg, Germany]) and incubated on ice for 30 minutes.

### Western blotting

The cell lysates (25–40 µg total protein or 15 µg nuclear protein per lane) were submitted to SDS- PAGE (on 12% gels), transferred to polyvinylidenediflouride membranes for Western Blot analysis. After blocking with 5% fat-free dried milk or BSA dissolved in TBS-T for 1 h at room temperature, membranes were incubated over night with antibodies raised against phosphorylated SAPK/JNK, p38, ERK, IκBα, IRF3 and total IRF3, SOCS2(Cell Signaling Technology, Beverly, MA) and p65NF-κB (BD Biosciences, Heidelberg, Germany) respectively according to the manufacturer's instructions. Binding of these primary antibodies was visualized with goat anti-rabbit/anti-mouse immunoglobulin coupled to horseradish peroxidase (Santa Cruz biotechnology, CA, USA). After stripping, the membranes were incubated and re-probed for new antibodies. Measurement of total ERK, IRF3 and β-actin protein served as loading controls.

### Statistical analysis

Statistical comparisons between groups were made by analysis of variance followed by T-test or Fisher's post-hoc test; p-values <0.05 were considered significant.

## Supporting Information

Table S1 Oligonucleotide sequences used for siRNA
(0.03 MB DOC)

Table S2 Oligonucleotide sequences used for real-time PCR
(0.05 MB DOC)
