# Supplementary material for: SOCS2 Influences LPS Induced Human Monocyte-Derived Dendritic Cell Maturation
Source: PLoS One. 2009 Sep 25;4(9):e7178. doi: 10.1371/journal.pone.0007178 (PMC2744869; doi:10.1371/journal.pone.0007178)
Supplement: Table S1 — Oligonucleotide sequences used for siRNA (0.03 MB DOC) [file pone.0007178.s001.doc]

**T**able S1. Oligonucleotide sequences used for siRNA

|  | Oligonucleotides (5’-3’) |
| --- | --- |
| hSOCS2 siRNA | Sense: AAC UAA UCU UCG AAU CGA AdTdT  Antisense: UUC GAU UCG AAG AUU AGU UdGdG |
| Negative control siRNA | Sense: UUC UCC GAA CGU GUC ACG UdT dT  Antisense: ACG UGA CAC GUU CGG AGA AdT dT |
