# Supplementary material for: SOCS2 Influences LPS Induced Human Monocyte-Derived Dendritic Cell Maturation
Source: PLoS One. 2009 Sep 25;4(9):e7178. doi: 10.1371/journal.pone.0007178 (PMC2744869; doi:10.1371/journal.pone.0007178)
Supplement: Table S2 — Oligonucleotide sequences used for real-time PCR (0.05 MB DOC) [file pone.0007178.s002.doc]

**T**able S2. Oligonucleotide sequences used for real-time PCR

|  | Oligonucleotides (5’-3’)^a^ |
| --- | --- |
| hCIS | F : AAC TGC CCA AGC CAG TCA T  R : CCC GAA GGT AGG AGA AGG TC |
| hSOCS1 | F : GAG AAC CTG GCT CGC ATC  R : AAC ACG GCA TCC CAG TTA AT |
| hSOCS2 | F : GAG CTC GGT CAG ACA GGA TG  R : AGT TGG TCC AGC TGA TGT TTT |
| hSOCS3 | F : GTC ACC CAC AGC AAG TTT CC  R : TCA CTG CGC TCC AGT AGA AG |
| hSOCS4 | F : CAC TCT TCA GGG CTT CCG TC  R : AGG CTA AAT CTG ATC GAG GTG G |
| hSOCS5 | F : ATC TGG AGA CAG CCA TAC CCA  R : CAA ATC AGG CAC GAG GCA GT |
| hSOCS6 | F : CCT AGT TGT CGC CCC AGA GA  R : CCC GTG GTG CCA ATC AA |
| hTNF-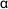 | F : CAC GCT CTT CTG CCT GCT G  R : GAT GAT CTG ACT GCC TGG GC |
| hIL-1β | F : ACA GAT GAA GTG CTC CTT CCA  R : GTC GGA GAT TCG TAG CTG GAT |
| hIL-6 | F : GAC AGC CAC TCA CCT CTT CA  R : AGT GCC TCT TTG CTG CTT TC |
| hCCL-4 | F : TGC GTG ACT GTC CTG TCT CT  R : TTC CTC GCG GTG TAA GAA A |
| hIFN-β | F : GAT TCC TAC AAA GAA GCA GCA A  R : CAA AGT TCA TCC TGT CCT TGA G |
| hCXCL-9 | F : GCA CCA ACC AAG GGA CTA TC  R: TGT TTG AAC TCC ATT CTT CAG TGT |
| hCXCL-10 | F : GAA ATT ATT CCT GCA AGC CAA T  R : CAG ACA TCT CTT CTC ACC CTT CT |
| RPII | F: GCA CCA CGT CCA ATG ACA T  R : GTG CGG CTG CTT CCA TAA |

^a^F, R indicate forward and reverse primers, respectively.
